# Pathobiological Implications of MUC16 Expression in Pancreatic Cancer

**DOI:** 10.1371/journal.pone.0026839

**Published:** 2011-10-31

**Authors:** Dhanya Haridas, Subhankar Chakraborty, Moorthy P. Ponnusamy, Imayavaramban Lakshmanan, Satyanarayana Rachagani, Eric Cruz, Sushil Kumar, Srustidhar Das, Subodh M. Lele, Judy M. Anderson, Uwe A. Wittel, Michael A. Hollingsworth, Surinder K. Batra

**Affiliations:** 1 Department of Biochemistry and Molecular Biology, University of Nebraska Medical Center, Omaha, Nebraska, United States of America; 2 Eppley Institute for Research in Cancer and Allied Diseases, University of Nebraska Medical Center, Omaha, Nebraska, United States of America; 3 Department of General and Visceral Surgery, Universitätsklinik Freiburg, Freiburg, Germany; 4 Department of Pathology and Microbiology, University of Nebraska Medical Center, Omaha, Nebraska, United States of America; The University of Kansas Medical Center, United States of America

## Abstract

MUC16 (CA125) belongs to a family of high-molecular weight O-glycosylated proteins known as mucins. While MUC16 is well known as a biomarker in ovarian cancer, its expression pattern in pancreatic cancer (PC), the fourth leading cause of cancer related deaths in the United States, remains unknown. The aim of our study was to analyze the expression of MUC16 during the initiation, progression and metastasis of PC for possible implication in PC diagnosis, prognosis and therapy. In this study, a microarray containing tissues from healthy and PC patients was used to investigate the differential protein expression of MUC16 in PC. MUC16 mRNA levels were also measured by RT-PCR in the normal human pancreatic, pancreatitis, and PC tissues. To investigate its expression pattern during PC metastasis, tissue samples from the primary pancreatic tumor and metastases (from the same patient) in the lymph nodes, liver, lung and omentum from Stage IV PC patients were analyzed. To determine its association in the initiation of PC, tissues from PC patients containing pre-neoplastic lesions of varying grades were stained for MUC16. Finally, MUC16 expression was analyzed in 18 human PC cell lines. MUC16 is not expressed in the normal pancreatic ducts and is strongly upregulated in PC and detected in pancreatitis tissue. It is first detected in the high-grade pre-neoplastic lesions preceding invasive adenocarcinoma, suggesting that its upregulation is a late event during the initiation of this disease. MUC16 expression appears to be stronger in metastatic lesions when compared to the primary tumor, suggesting a role in PC metastasis. We have also identified PC cell lines that express MUC16, which can be used in future studies to elucidate its functional role in PC. Altogether, our results reveal that MUC16 expression is significantly increased in PC and could play a potential role in the progression of this disease.

## Introduction

Pancreatic cancer (PC) is an extremely lethal malignancy. According to the American Cancer Society, the estimated number of new cases and deaths due to PC in the United States in 2010 were 43,140 and 36,800 respectively with a 5 year survival rate of 6% [1]. Adenocarcinoma of the pancreatic ducts accounts for nearly 95% of all pancreatic tumors [2] and is associated with a median survival of only 3–6 months. The poor outlook of PC patients is attributed in large part to the clinically silent nature of this malignancy which often leads to its diagnosis at an advanced and often unresectable stage of the disease.

Several proteins have been reported to be dysregulated during the initiation and progression of PC. One such family of proteins whose expression is aberrantly upregulated in PC is mucins. These are high molecular weight, heavily glycosylated proteins that serve several functions in normal tissues including lubrication and entrapment of harmful pathogens [3]–[7]. Their expression is apparently altered in several malignant conditions including PC [8].

CA125 (MUC16) is a cell surface glycoprotein that was first identified by Bast *et al* in 1981[9]. MUC16 is cleaved and shed into the bloodstream and has been the focus of active research as a biomarker in the serum for a variety of tumor types [10]. It is currently the only serum tumor marker routinely used in clinics for the diagnosis and particularly predicting prognosis in ovarian cancer patients [11]. CA125 is the best known antibody that recognizes MUC16 both in tissues and in body fluids and is targeted to an epitope located in the tandem repeat region of the MUC16 protein [4], [12].

Structurally the MUC16 protein comprises of an extracellular N-terminal domain consisting of more than 22,000 amino acid residues and is believed to be heavily glycosylated. The central domain contains up to 60 glycosylated peptide sequences repeated in tandem (a characteristic feature of the mucin family) followed by a C-terminal domain containing a potential proteolytic cleavage site, a transmembrane domain and a short cytoplasmic tail with several potential sites of phosphorylation [13], [14].

In the present study, we have analyzed the expression of MUC16 in PC tissues and cell lines using the CA125 monoclonal antibody. Further we have analyzed the expression of its mRNA in tissues isolated from PC and pancreatitis patients by RT-PCR. The overall objective of our study was to investigate whether there is a differential expression of MUC16 during the progression and development of PC and to examine a possible correlation between MUC16 expression and tumor characteristics. Our study suggest that MUC16 is not expressed in the normal pancreatic ducts but upregulated during PC progression and development, thus suggesting a potential role for MUC16 in PC pathogenesis and its clinical diagnosis.

## Materials and Methods

### Ethics Statement

A written informed consent was obtained for all non-archival tissue prior to tissue collection for all samples obtained through UNMC.

### Tissue specimen and cell lines

Fifty-eight PC and 8 normal pancreatic tissue samples (formalin fixed and paraffin-embedded) were obtained from Accumax (Array number A207IV). The array contained tissues classified as non-neoplastic (8 spots), well-differentiated adenocarcinoma (12 spots), moderately differentiated adenocarcinoma (22 spots) and poorly differentiated adenocarcinoma (24 spots). Expression of MUC16 was analyzed by RT-PCR from 2 normal human pancreatic tissues, 6 pancreatitis and 17 pancreatic cancer tissues which were obtained after approval of the protocol by the IRB (IRB- 491-97) at the University of Nebraska medical Center, Omaha, NE. The mRNA was converted to cDNA using oligo dT primers. The primers used to check for MUC16 expression are MUC16_F-5′ GTCCCCAACAGGCACCACACCG-3′ and MUC16_R-5′GGGCACTGTTGCTGGACGTTGTATT-3′ and the PCR product was sequence verified at the UNMC DNA sequencing facility.

Further, formalin fixed and paraffin embedded (FFPE) PC tissue samples from 34 PC patients comprising of normal pancreas (7 spots), primary PC (31 spots) and metastasis to the liver (23 spots), lungs (11 spots), lymph node (17 spots) and omentum/diaphragm (11 spots) obtained from University of Nebraska Medical Center's rapid autopsy program (IRB-091-01) were also analyzed to investigate the change in MUC16 expression during PC metastasis. Under the rapid autopsy program at UNMC, tissues from donor patients are harvested within three hours after their death and the specimens flash frozen in liquid nitrogen or placed in formalin for immediate fixation. Tissue microarrays (TMAs) made from paraffin blocks of tissues from the rapid autopsy program were used for MUC16 immunostaining. In addition to the tumor cores, each block contained control specimens from the non-neoplastic colon, kidney and tumor adjacent pancreas from the same donors. The TMA blocks were cut into 4 µM sections and mounted on charged slides.

Twenty-five PC tissue samples containing Pancreatic Intraepithelial Neoplasms (PanIN) lesions of varying grades (Number of lesions identified-PanIN I- 163; PanIN II-197; PanIN III-26 and normal ducts-140) adjacent to the areas of PC were also obtained after approval of the protocol by the Institutional Review Board (IRB-491-97) at the University of Nebraska Medical Center, Omaha, NE. Four micron thick paraffin sections were cut and stained with hematoxylin and eosin for pathological evaluation. The grade of PanINs and MUC16 expression in each type of PanIN lesion was assessed by the surgical pathologist (S.M.L).

The expression of MUC16 mRNA and protein was also analyzed in a panel of PC cell lines (MiaPaca, Panc89, DanG, HPAC, SU86.86, Colo357, CD18/HPAF, HUPT3, Capan1, Suit2, CD11, T3M4, FG, Aspc1, Panc1, HG625, Capan2 and BxPC3) using primers previously mentioned. The cell lines were grown at 37°C in presence of 5% CO_2_ in DMEM supplemented with 10% fetal calf serum and antibiotics (penicillin and streptomycin 100 µg/ml). All the cell lines were obtained from ATCC.

### Immunohistochemistry

The slides were processed for immunostaining as described previously [15], [16]. The anti- MUC16 mouse monoclonal antibody (M11 clone, manufactured by Dako, Carpinteria, CA, USA) was used as the primary antibody (Stock: 764 µg/ml dilution factor 1∶500).

All stained slides were scored by a pathologist under a Nikon E400 Light Microscope and representative photographs taken. Staining intensity for MUC16 (CA125) was scored on a scale of 0–3 (0-negative, 1-weak, 2-moderate, 3-strong immunoreactivity). The percentage of cells positive for MUC16 within a given lesion was scored on a scale of 1–4 as follows: 1: 0–25% cells positive; 2: 26–50% positive; 3: 51–75% positive; and 4: 76–100% positive. The score of the staining intensity and the percentage of immunoreactive cells were then multiplied to obtain a composite score ranging from 0 to 12. A section was considered “positive” for MUC16 if the intensity of MUC16 was >1. Accordingly tissues were also classified as being “positive” or “negative” for MUC16 expression.

### Confocal immunofluorescence microscopy

PC cells were processed for confocal microscopy as described previously [17], [18]. Briefly, cells were grown at 37°C for 48 h on sterile glass cover slips, washed with Hanks buffer containing 0.1 M HEPES and fixed in ice-cold methanol at 20°C for 2 min. Cells were blocked with 10% goat serum in phosphate buffered saline (PBS) for 30 min, followed by incubation with anti-MUC16 monoclonal antibody (CA125) diluted in PBS (CA125 stock: 764 µg/ml; dilution factor 1∶500) for 1 h at room temperature. Cells were washed for 10 min (×4 times) with PBS and then incubated with FITC-conjugated goat anti-mouse secondary antibody for 30 min. Cells were again washed (10 min ×4) and mounted on glass slides in anti-fade Vectashield mounting medium (Vector Laboratories, Burlingame, CA, USA).

### Western blot analysis

PC cell lines were processed for protein extraction and was followed by western blotting by SDS–agarose as previously described [17], [18]. Heat denatured lysates were resolved on a 2% SDS–agarose gel by electrophoresis and subsequently transferred on to PVDF membranes. After transfer the membrane was blocked with 5% nonfat dry milk in PBS for 2 h, and incubated with anti-MUC16 mAb (stock:764 µg/ml dilution factor 1∶1000) or anti-β-actin monoclonal antibody overnight at 4°C. The membranes were washed with PBS containing 0.1% tween-20 and subsequently incubated with horseradish peroxidase- conjugated goat anti-mouse secondary antibodies (diluted 1∶2000 in 5% nonfat dry milk in PBS) (Amersham Biosciences Buckinghamshire, UK) for 1 h. The signal was detected using enhanced chemiluminescence (ECL, Amersham Biosciences, Buckinghamshire, UK).

### RNA Extraction and RT-PCR

Total RNA was isolated from tissues using the *mir*Vana miRNA isolation kit (Ambion, Foster city, CA, USA) and from cell lines using the QIAGEN RNeasy Mini kit (QIAGEN, Valencia, CA, USA) according to the manufacturer's protocol. The mRNA isolated was converted to cDNA using oligo dT primers. The cDNA diluted 1∶5 was used to determine the expression of MUC16 mRNA using PCR according to previously described protocol [19]. The products were run on a 1.5% agarose gel containing ethidium bromide (10 µg/ml). The primers used to check for MUC16 expression are those that have been previously mentioned.

### Statistical analysis

Continuous variables (e.g.composite score) were compared using a Student's two-tailed t-test assuming unequal variance. Categorical variables (stage, grade of tumor, organ of distant metastasis) were compared using the Kruskal Wallis ANOVA test or the Fisher's exact test. A p-value<0.05 was considered to be statistically significant. All statistical analysis was done using Medcalc for Windows version 9.6.4.0 software (MedCalc software bvba, Mariakerke, Belgium).

## Results

### MUC16 is differentially overexpressed in pancreatic adenocarcinoma tissues

**Figure 1:**
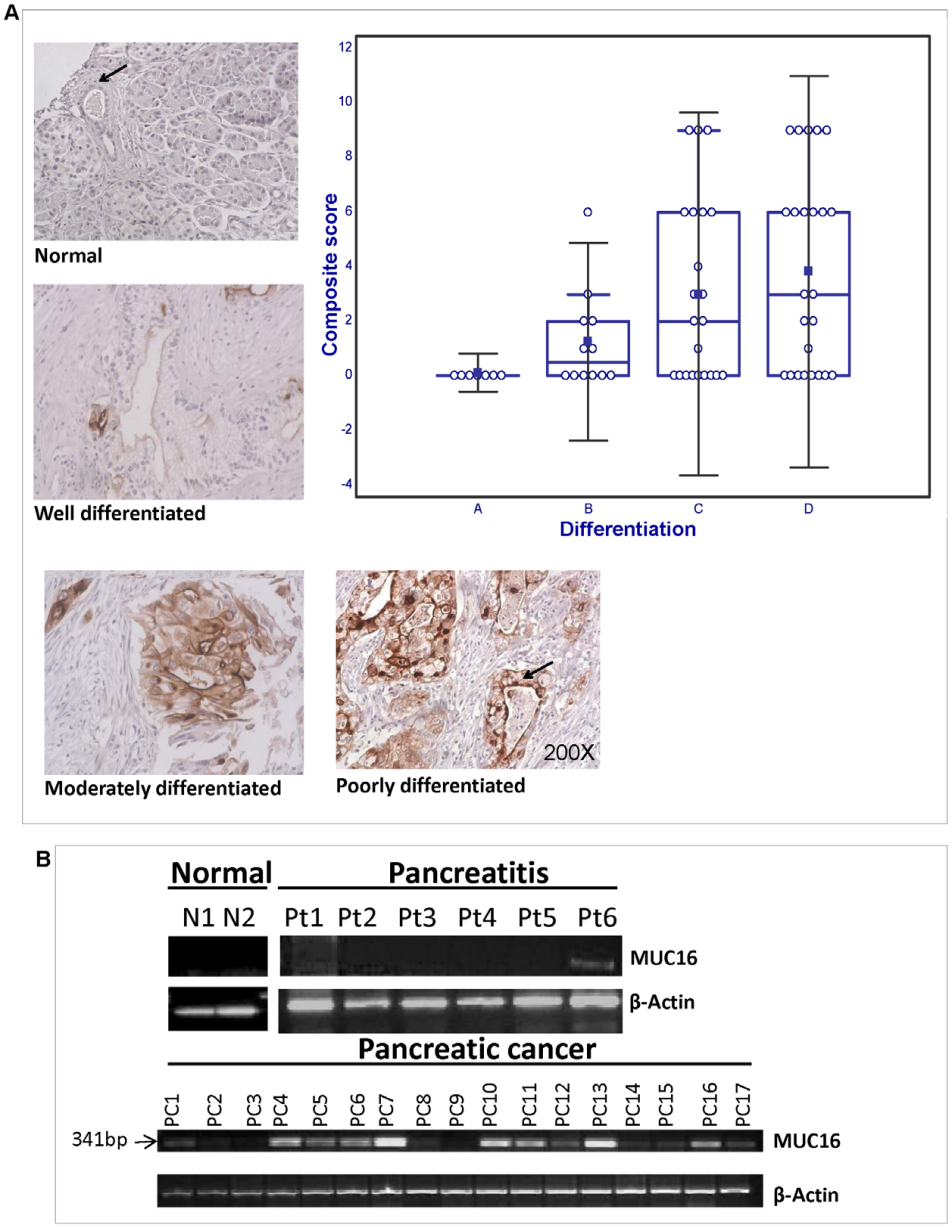
The expression of MUC16 in normal (N) and pancreatic cancer (PC) tissues by immunohistochemistry. The tissue sections were obtained from ACCUMAX in the form of an array and were stained with anti-MUC16 monoclonal antibody. The stained sections were observed under the microscope and the immunoreactivity was judged by the intensity and spread of the dark stain. Anti-MUC16 antibody showed no staining in the normal pancreas tissue (both ducts and acini) while a strong staining was observed in the cancerous tissues. (A) Box plot representing the score of MUC16 across the various grades of PC. From the box plot we observed the immunoreactivity to be higher in the poorly (D) and moderately differentiated (C) tissues in comparison to well differentiated (B) tissues. The normal pancreas (A) tissue is also negative. Representative sections demonstrating MUC16 expression in normal pancreatic ducts and various grades of invasive adenocarcinoma are shown. Note the predominant membrane staining of MUC16 in PC. (B) Expression studies of MUC16 in normal, pancreatitis and pancreatic cancer tissues by RT-PCR. RT-PCR was performed on mRNA isolated from normal human pancreatic tissue (N1, N2), human pancreatitis tissue (Pt1–Pt6) and human pancreatic cancer tissue (PC1–PC17). No amplification was observed in the normal tissues but amplification was observed in the pancreatic cancer and pancreatitis tissues. Actin was used as an internal control.

To identify the expression pattern of MUC16 in PC pathogenesis, its expression was compared between non-neoplastic ducts and pancreatic adenocarcinoma using a tissue microarray comprising non-neoplastic pancreas (n = 8) and tissues from primary PC of varying grades (n = 58). A tissue sample was considered to be positive if at least >5% of the cells expressed MUC16. MUC16 expression was not observed in the non-neoplastic ducts (Figure 1A). However, in 38/58 (65%) cases of PC, the malignant ducts were positive for MUC16. The expression of MUC16 was significantly higher in PC when compared to the non-neoplastic ducts (p = 0.003 by the two-tailed Fisher's exact test). Further, to determine whether there is a variation in MUC16 expression with the progression of PC, we compared its expression between PC tissues classified by tumor stage and grade. There was a progressive increase in the expression of MUC16 with loss of tumor differentiation, with 50% of the well-differentiated (6/12), 59% of the moderately differentiated (13/22) and 66% of the poorly differentiated PC tissues (16/24) being positive (Figure 1A). While the expression of MUC16 was not significantly different between the three groups, the mean composite score was significantly higher in moderate and poorly differentiated PC compared to well-differentiated adenocarcinomas (p = 0.02 and 0.001 respectively). However, the composite score was not significantly different between moderate and poorly differentiated PC cases. This suggests that MUC16 expression increases significantly with loss of differentiation of PC tissues.

The differential expression of MUC16 in PC was also examined by checking the expression of MUC16 at the transcriptional level by isolating mRNA from normal human pancreatic, pancreatitis and PC tissues. MUC16 mRNA expression was absent in normal pancreatic tissues (0/2, 0%) but was present in 12/17 (71%) PC and 1/6 (16.7%) pancreatitis tissues (Figure 1B). Most of the PC tissues were classified as moderate to poorly differentiated PC (8/17). There was 1 case of well differentiated PC, one of pseudopapillary neoplasm and one each of a mucinous cystic adenocarcinoma and giant cell tumor. 5/8 (62.5%) of the moderate-poorly differentiated, 1/1 (100%) of well differentiated, 1/1 giant cell tumor and 1/1 of mucinous cystic adenocarcinoma expressed MUC16. Six PC patients also had a history of chronic pancreatitis (CP). 5/6 (83%) of these tumors were also positive for MUC16. In comparison, 7 PC patients did not have any history of CP. Of these cases, 5/7 (71.4%) were positive for MUC16. The expression of MUC16 was not significantly different between those with or without a history of CP (Table 1).

**Table 1:** Information of PC patients from whom tissues were isolated to study MUC16 expression.

Sample Number	Age	Gender	Location of tumor on pancreas	Tumor differentiation grade	Chronic Pancreatitis	MUC16 expression
1	73	M	Head	Moderately differentiated PDAC	No	+
2	83	M	Neck	Moderately differentiated PDAC	No	−
3	16	F	Tail	Solid pseudopapillary neoplasm		−
4	62	M	Tail	N/A	No	++
5	60	F	Head	Well differentiated	Yes	+
6	80	M	Head	Moderate to poorly differentiated adenocarcinoma	Yes	+
7	46	M	N/A	Moderately differentiated adenocarcinoma	Yes	+++
8	74	F	Body and Tail	Poorly differentiated PDAC	Yes	−
9	70	F	Head	N/A	No	−
10	58	M	Tail	Moderate to poorly differentiated adenocarcinoma	No	++
11	59	M	Tail	Giant cell tumor	Yes	++
12	35	F	Head and neck	Mucinous cyst adenocarcinoma	No	+
13	72	M	Head	Moderate to poorly differentiated adenocarcinoma	Yes	+++
14	N/A					
15	69	M	N/A	Poorly differentiated adenocarcinoma	No	+
16	N/A					
17	N/A					

Abbreviations: PDAC-Pancreatic Ductal Adenocarcinoma; N/A-Not Available.

### Differential upregulation of MUC16 in high grade pancreatic dysplasia

**Figure 2:**
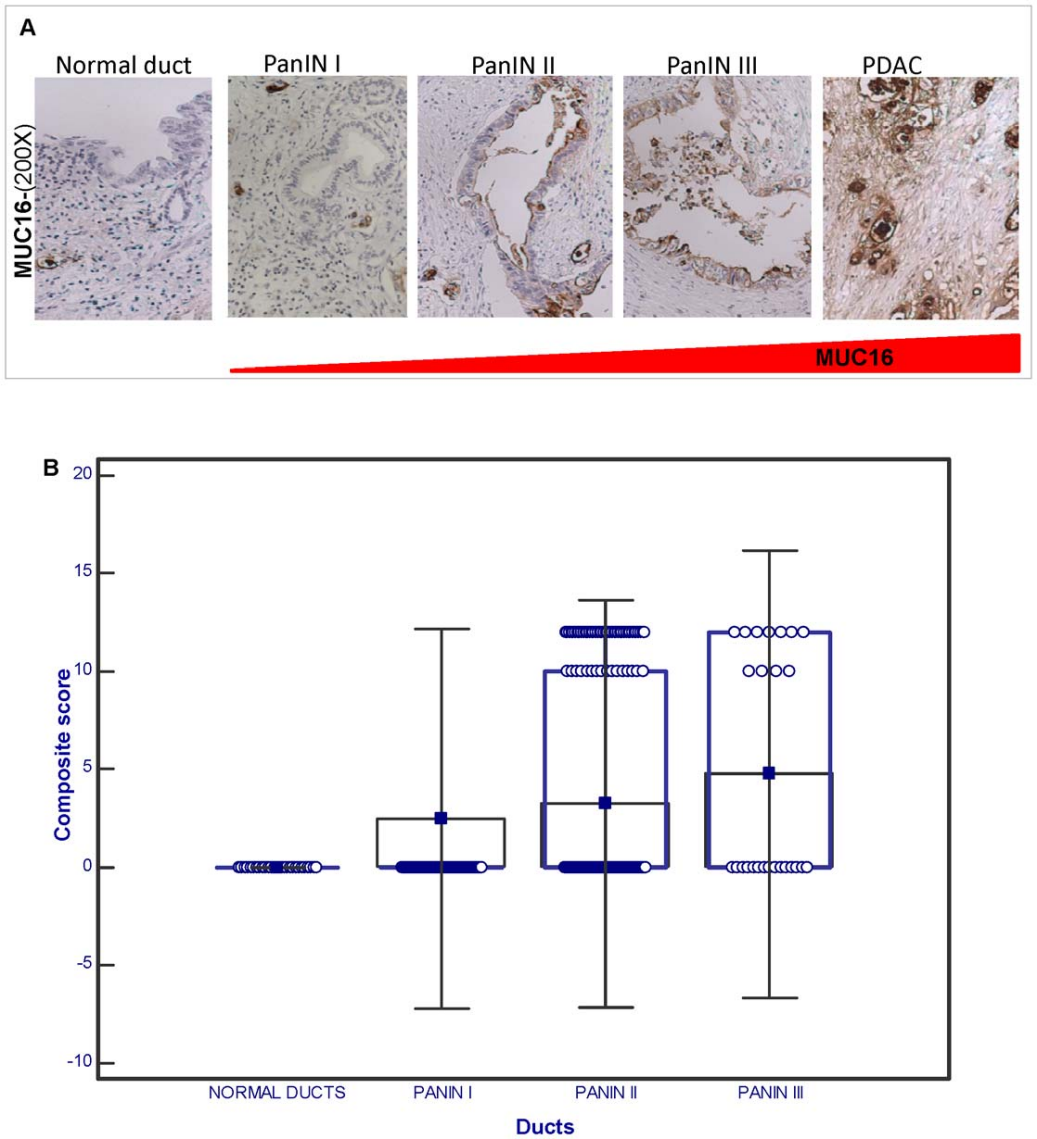
Expression of MUC16 in PanIN lesions and normal ducts. (A) MUC16 expression was evaluated in tissues containing both the normal pancreas, and adjacent dysplastic lesions. While MUC16 expression was weak in the low-grade, early stage PanIN lesions (PanIN I), it progressively increases with increasing dysplasia with the highest expression observed in high-grade dysplasia (PanIN III) and PC. Note the predominant membrane staining of MUC16 in all grades of PanINs (Original magnification ×200). (B) Box plot representing the composite score of MUC16 across the different grades of PanIN lesions and normal ducts. From the box plot we observe that MUC16 is strongly expressed in PanIN II and III when compared to PanIN I.

Having observed that MUC16 is aberrantly expressed in pancreatic ductal adenocarcinoma we next sought to study the expression of MUC16 during the development of PC. For this, we studied its expression in pre-malignant lesions known to precede invasive adenocarcinoma, termed as pancreatic intraepithelial neoplasia (PanINs). PanIN lesions are classified as PanIN I, PanIN II and PanIN III which correspond to low, intermediate and high grade dysplasia and are characterized by well-defined histological changes including nuclear atypia, nuclear crowding, pseudostratification and in high grade PanINs, cribriforming [20]. We observed that 20% of PanIN-I (33/163), 28% of PanIN-II (55/197) and 42% of PanIN-III lesions (11/26) were positive for MUC16 expression but its expression was not detected in the adjacent normal ducts (n = 140) as shown in Figure 2A. MUC16 expression was significantly higher in all three stages of dysplasia (p<0.00001) compared to the normal ducts. But MUC16 expression was significantly higher in high grade dysplasia (PanIN-III) compared to low-grade dysplasia (PanIN-I, p = 0.02). However, there was no significant difference in MUC16 positivity between PanIN-I and PanIN-II (Figure 2B). Like in invasive carcinoma, MUC16 predominantly localized to the cell membrane of the dysplastic cells.

### Comparison of MUC16 expression between primary and metastatic pancreatic cancer

**Figure 3:**
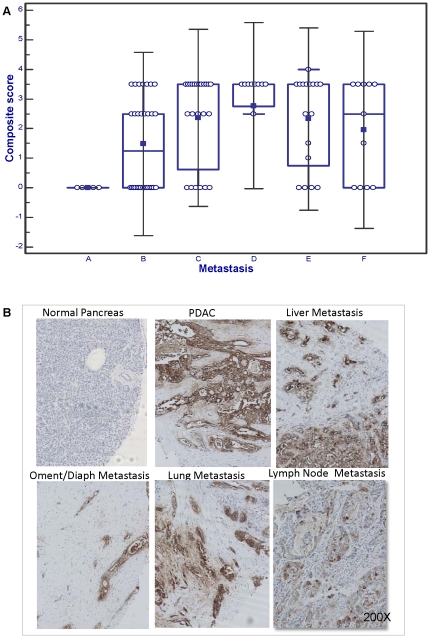
Expression of MUC16 in matched primary and metastatic pancreatic cancer tissues. To investigate the alteration in MUC16 expression with progression, we investigated the expression of MUC16 in matched primary pancreatic cancer and metastasis to either the lung, lymph node, liver or the omentum/diaphragm. In (A) while the expression of MUC16 was higher in metastatic PC than in the primary tumor, this was not significant. A- Normal pancreas; B- pancreatic cancer; C- Liver metastasis; D- Lung metastasis; E- Lymph Node metastasis; F- Omentum/Diaphragm metastasis. Further, (B) shows that in the same patient, the non-neoplastic ducts were negative, while there was a strong expression of MUC16 in the primary pancreatic tumor and this was maintained even in the metastasis.

Several proteins are known to have a differential expression in primary vs. metastatic cancer [21], [22]. To investigate whether the expression of MUC16 is altered during the metastasis of PC to distant sites, we investigated its expression in matched (obtained from the same patient) primary pancreatic adenocarcinomas and metastasis to the lymph nodes, lungs, liver and omentum/diaphragm (obtained as part of the rapid autopsy program). MUC16 was not expressed in any of the non-neoplastic ducts, while the primary and metastatic tumors from the same patient expressed MUC16 with nearly the same intensity. The results are summarized in Figure 3A and 3B. There was no significant difference in the composite score between the primary and metastatic sites (summarized in the box plot). However, patients who expressed MUC16 in their primary tumor also expressed MUC16 at the metastatic sites. This suggests that pancreatic tumors maintain MUC16 expression during their spread, possibly pointing to the role of MUC16 in PC cell dissemination.

### Expression of MUC16 in the various human pancreatic cancer cells

**Figure 4:**
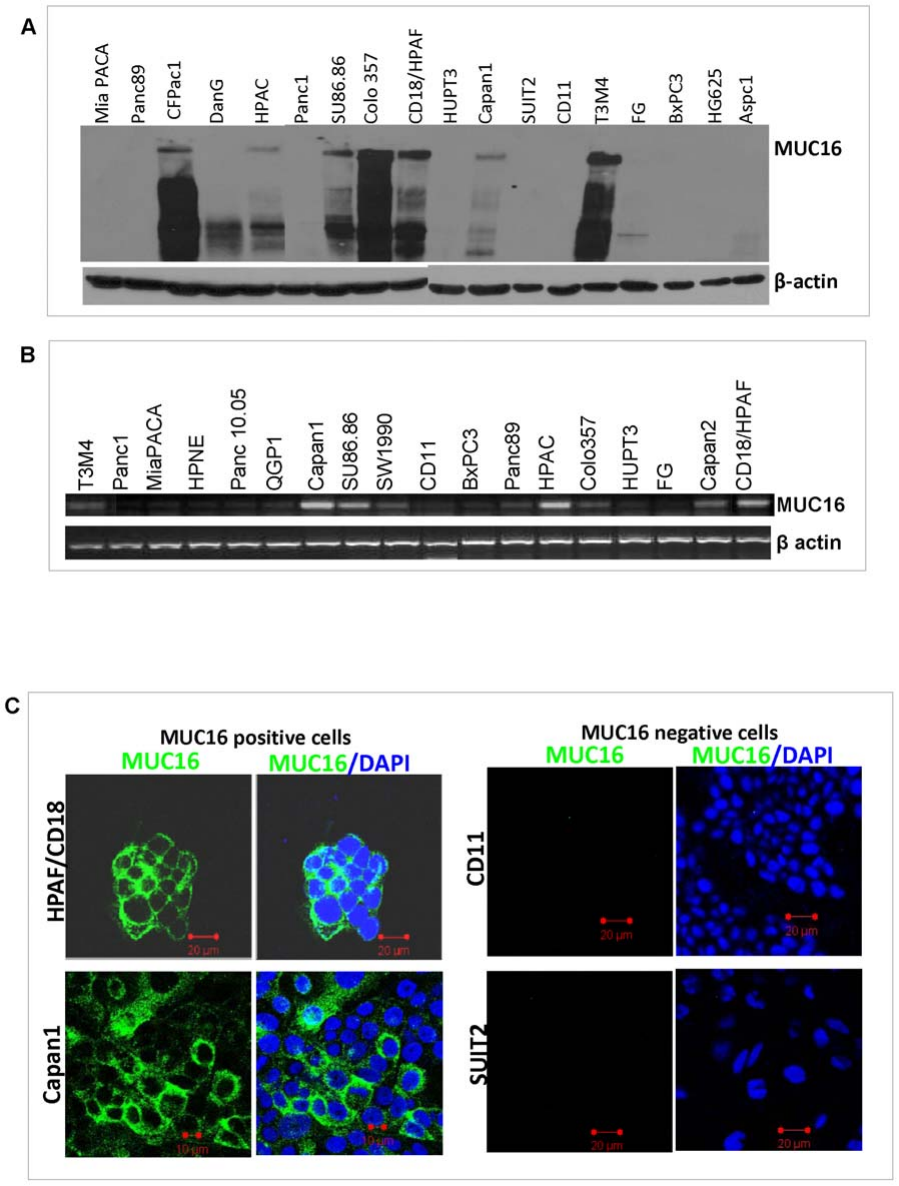
Expression of MUC16 in a panel of PC cell lines using western blot, RT-PCR and confocal studies. (A) Western blot analysis of MUC16 expression in PC cell lines. Protein lysates from eighteen PC cell lines were resolved on a 2% SDS-agarose gel. MUC16 expression was observed in DanG, HPAC, SU86.86, Colo357, CD18/HPAF, Capan1 and T3M4 cell lines. β-actin was used as an internal control (B) RT-PCR analysis of MUC16 expression in various PC cell lines. Actin was used as an internal control. (C) Immunofluorescence studies of two cell lines (CD18 and Capan1) that expresses MUC16 and two cell lines (CD11 and SUIT2) that do not express MUC16.

Having demonstrated that MUC16 was differentially expressed in PC tissues, we further investigated its expression in PC cell lines. Expression of MUC16 both at the mRNA (PCR product size is 341 bp) and protein level were observed in Colo357, T3M4, HPAF/CD18, DanG, HPAC, SU86.86, FG and Capan-1 cells (Figure 4A and 4B). All other PC cell lines tested were negative for MUC16 expression. To delineate the subcellular localization of MUC16, immunofluorescence studies were performed in Capan1 and HPAF/CD18 cells (MUC16 expressing) and SUIT2 and CD11 (MUC16 non-expressing) cells. Staining was noted in both the positive cell lines concordant with the distribution of MUC16 in the tissues and no staining was observed in the negative cell lines (Figure 4C).

## Discussion

PC is the fourth leading cause of cancer related deaths in the United States with only about 20% of the patients surviving for 2 years and only 6% for five years. Its incidence to mortality ratio has remained virtually unchanged for the past several decades despite a considerable understanding of its biology [23], [24]. This high mortality rate among PC patients is due to the tendency of the cancer cells to metastasize early. This, together with the poor response to therapy and high-rate of recurrence makes it one of the most lethal cancers known to man [24]. PC is often diagnosed at an advanced stage, chiefly due to the lack of reliable early diagnostic markers [24]. Therefore, there is an urgent need to identify specific early detection marker(s) and suitable molecular targets to combat PC. Mucins are one of the major biomarkers that have emerged in recent years as highly specific diagnostic markers in several malignancies including pancreatic, gynecologic and aerodigestive tract malignancies [25]. Further, their aberrant expression has been demonstrated to modulate cell growth, differentiation, transformation, adhesion, invasion and immune surveillance [7].

CA125/MUC16 is a tumor biomarker that is currently used for the follow-up of patients with ovarian cancer [26]. However, its role in PC pathogenesis remains unexplored. In the present study, we examined the expression pattern of MUC16 in PC tissues and compared it with that in the normal pancreas. Further, we also studied its association with PC development and with tumor stage, grade and metastasis. Interestingly, our results showed that the normal pancreas does not express MUC16 but its expression is significantly upregulated in 12/17 PC and 1/6 pancreatitis tissue. It has been previously shown that there is an association between the carcinoma of the pancreas and chronic pancreatitis (sporadic and familial) and the standardized incident ratio of developing PC in chronic pancreatitis patients is 14–18 [27]. This observation of MUC16 being significantly upregulated in PC is coherent with the expression of other membrane bound mucins MUC4 and MUC1 which are also aberrantly expressed in PC and have been identified as potential diagnostic markers for this malignancy [24], [28]–[31] We also observed that MUC16 expression increased progressively with loss of differentiation of the PC tumor. It has been previously observed that PC patients who have been diagnosed with distant metastasis had the tendency to have poorly differentiated pancreatic adenocarcinoma [32], [33]. Thus, our findings suggest that MUC16 may have an important role to play in the progression and metastasis of PC.

According to the well-known progression model for PC, ductal carcinoma develops from non-neoplastic ducts though a series of pre-malignant lesions termed as PanINs [33]. We observed that MUC16 expression increases progressively from PanIN I to PanIN III. Particularly, the percentage of MUC16 positivity in high-grade PanIN lesions was significantly higher than that of low-grade PanINs. This suggests that MUC16 expression is altered at the stage of pancreatic dysplasia and may play a critical role in the progression of PC. MUC4, another membrane bound mucin has been previously shown to be differentially upregulated with progressively increasing dysplasia, suggesting the possibility that there may be certain common regulatory pathways that modulate the expression of both these mucins during PC development [28], [34], [35]

The high mortality rate in PC patients is due to the frequent occurrence of distant metastasis. In an analysis of 4,012 autopsies performed on PC patients between 1914 and 1943 it was reported that the most common site of distant metastasis was the liver, followed by the peritoneum, lung and pleura, bones and the adrenal glands [33], [36]. But PC is not limited to these organs. Even small PCs (<2 cm in diameter) exhibit metastasis, supporting the premise that PC is a malignancy that metastasizes very early during its progression [33], [37] Among the several molecules observed to play a role in the metastasis of PC cells, mucins have emerged as one of the key determinants. We have previously shown that MUC4, another transmembrane mucin when expressed promotes metastasis and invasiveness in PC cells [18], [38], [39]. In the present study, we observed that those primary PCs that express MUC16 also express MUC16 with nearly equal intensity in the metastatic sites. This suggests that PC cells may maintain their expression of MUC16 during the metastatic process. MUC16 has been previously demonstrated to be important in the metastasis of solid tumors to the central nervous system via its interaction with mesothelin, a protein differentially expressed in normal mesothelial cells, mesotheliomas and some other mesenchymal malignancies [40], [41]. Hence it is possible that MUC16 might interact with mesothelin and facilitates metastasis in PC.

The molecular mechanisms driving metastasis in PC requires a better understanding of proteins that modulate epithelial-mesenchymal transition (EMT) and the reverse process (MET), which are necessary for the detachment and re-attachment of tumor cells at the site of metastasis respectively. The results of our study suggest that MUC16 might have a role for the development and progression of PC and studying its specific role in the progression of PC will be the basis of our next study. Its upregulation, which was particularly strong during the late stages of PC dysplasia, suggests that the mechanisms that turn on its expression are possibly turned on late during PC development. Studies on MUC4 mucin have revealed that its expression is silenced in normal ducts by virtue of hypermethylation of its promoter [42], [43]. Whether similar epigenetic changes also regulate MUC16 expression remains to be examined in future studies. The detection of MUC16 in high-grade PanINs (considered to be the true dysplastic lesions with a high risk for invasive cancer) suggests its potential use in the early detection of potentially malignant lesions in the pancreas. MUC16 is also shed in the bloodstream (known as CA125), making it an attractive molecule for investigation as a potential secreted biomarker for PC [44].

Further to identify a suitable *in vitro* model to investigate the functional role of MUC16, a panel of PC cell lines was screened for MUC16 expression both at the protein and the mRNA level. On performing western blot analysis, it was observed that MUC16 expression either appeared as a single band or as a streaky band. It has been previously shown that when mucins are treated with 2-mercaptoethanol and analyzed on a SDS-PAGE gel, a streaky band is obtained as the mucins have been reduced from its oligomeric structure to its monomeric form. This monomeric form enables mucins to migrate faster on the gel and the intact oligomers remain in the well [45]. This thus explains the differential expression pattern of MUC16 obtained across the various PC cell lines screened. In addition, we also observed that MUC16 expressing cell lines, such as Capan 1 (liver met), Colo 357 (lymph node met) and T3M4 (lymph node met) were derived from metastatic sites while the MUC16 non expressing cell lines such as Panc1, AsPC1 and BxPC3 were isolated from the primary tumor site (pancreas). Further, from the RT-PCR studies we observed that the mRNA levels of some PC cell lines did not corroborate with their corresponding protein levels. We speculate that MUC16 mRNA undergoes post transcriptional processing in certain cell lines. We are currently performing preliminary studies to further investigate this hypothesis.

In conclusion, our study shows that MUC16 is expressed only in pancreatic adenocarcinomas when compared to undetectable levels in the normal pancreas. The expression of MUC16 is stronger with progressive worsening pancreatic dysplasia (from PanIN I lesion to PanIN III). A strong expression pattern of MUC16 was observed in matched primary tumors and metastatic tumors at all the sites examined (liver, lung, lymph nodes and omentum/diaphragm) suggesting that MUC16 could be playing an important role in the progression and metastasis of PC. Further, MUC16 expression was observed in several PC cell lines at both the protein and the mRNA level. Overall, these results suggest a potential implication of MUC16 in PC pathogenesis and provide a basis for future studies aimed at unraveling the functions of this large membrane bound glycoprotein in PC.
